# Age, Sex, and Race/Ethnicity in Clinical Outcomes Among Patients Hospitalized With COVID-19, 2020

**DOI:** 10.3389/fmed.2022.850536

**Published:** 2022-05-12

**Authors:** Jusung Lee

**Affiliations:** Department of Public Health, University of Texas at San Antonio, San Antonio, TX, United States

**Keywords:** COVID-19, clinical outcomes, age, race, sex

## Abstract

The COVID-19 pandemic revealed the disproportionate risk of poor clinical outcomes among population subgroups. The study investigates length of stay (LOS), intensive care unit (ICU) admission, and in-hospital death across age, sex, and race among patients hospitalized with COVID-19. A pooled cross-sectional study analyzed hospital discharge data of state-licensed hospitals in Texas from April to December 2020. Of 98,879 patients, males accounted for 52.3%. The age distribution was 31.9% for the 65–79 age group, 29.6% for those aged 50–64, and 16.3% for those older than 79. Whites constituted the largest proportion (42.6%), followed by Hispanics (36.2%) and Blacks (13.1%). Higher in-hospital death rates were found among patients aged 80 and over (Adjusted Risk Ratio (aRR) 1.12, 95%CI 1.11–1.13) and patients aged 65–79 (aRR 1.08, 95%CI 1.07–1.09) compared to patients aged 19 and below. Hispanics (aRR 1.03, 95%CI 1.02–1.03) and other minorities (aRR 1.02, 95%CI 1.02–1.03) exhibited higher in-hospital death rates than whites, and these patients also had longer LOS and higher ICU admission rates. Patients aged 65–79, 50–64, and 80 and over all had longer hospital stays and higher ICU admission rates. Males experienced poor health outcomes in all assessed outcomes. Findings showed that disparities in clinical outcomes among population subgroups existed and remained throughout 2020. While the nation has to continue practicing public health measures to minimize the harm caused by the novel virus, serious consideration must be given to improving the health of marginalized populations during and beyond the pandemic.

## Introduction

Ever since the first case of the severe acute respiratory syndrome coronavirus 2 (SARS-CoV-2) was confirmed in the United States, the COVID-19 pandemic has disrupted the lives of every American. One troubling feature of the public health crisis caused by the pandemic is the excess harm posed to marginalized and vulnerable populations, which has punctuated the national awareness of health disparities between population subgroups (1).

The unprecedented global pandemic has revealed the disproportionate risk of poor clinical outcomes among population subgroups. Age has been suggested as a strong predictor of mortality—that is, the risk of mortality from COVID-19 increases with age (2, 3). Older adults have been identified as the most vulnerable group to the effects of the pandemic. Also, studies have reported that males are at a disproportionate risk of severe conditions and death caused by COVID-19 (4, 5). In the middle of the pandemic, the country was also exposed to racial/ethnic health disparities, which prompted a harsh national public health discourse. Studies, including Louisiana reports, have found significantly higher hospital admissions, intensive care unit (ICU) admission or severe illness, and in-hospital mortality among racial minorities compared to their white counterparts (6–9).

So far, studies on hospitalized patients often relied on data from a single or a few healthcare systems. Previous studies may exhibit a limitation in interpreting findings to a larger group of the patient population hospitalized with COVID-19. This study uses data on hospitalized patients with COVID-19 from all state-licensed hospitals in Texas except those that are statutorily exempt from reporting requirements. The inclusion of a large number of hospitals furthers representative evidence of hospitalized patient population and improves generalizability. Second, less is known about changing clinical outcomes across population subgroups. This study provides insight into the changes in clinical outcomes over the three quarters of 2020 across key demographic characteristics among patients with COVID-19. Also, Texas, one of the states hardest hit by COVID-19, has unique demographics, with a larger Hispanic or Latino population (39.7%) compared to the national average (18.5%). The study enhances the understanding of clinical outcomes in Texas, and how they vary from national trends.

The aim of the study is to investigate differences in length of stay (LOS), intensive care unit (ICU) admission, and in-hospital death across age, sex, and race/ethnicity and to examine how the variations change over time in 2020 using Texas inpatient discharge data.

## Methods

### Study Design and Data

The pooled cross-sectional study used the de-identified public-use data of Texas hospital discharge for the last three quarters in 2020. The hospital discharge data from all state-licensed hospitals in Texas except those that are exempt from the reporting requirement contains patients' demographics and healthcare information related to hospitalization. The three quarterly inpatient discharge files were merged and then were linked with the 2013 Urban-Rural Classification Scheme from the National Center for Health Statistics using patients' residential county. The study patients were identified through confirmed COVID-19 (U07.1) using the International Classification of Disease, 10th revision, Clinical Modification (ICD 10-CM) diagnosis code, following the US Centers for Disease Control and Prevention's Official Coding and Reporting Guidelines (10). The analysis included 98,879 patients after excluding missing (2.8%) on any variables in the study.

### Measurement

#### Outcomes

In-hospital death was a primary outcome of interest, capturing patients' expiration at the hospital. LOS and ICU admission were also outcomes of interest. In-hospital death and ICU admission were dichotomized, and LOS was treated as a count variable.

#### Independent Covariates

Age, sex, and race/ethnicity were key independent variables based on the literature review (7, 11). Age was categorized: below 20, 20–34, 35–44, 45–55, 55–64, 65–79, 80 and over. Sex was male and female. Patients' self-reported race/ethnicity was constructed using race and ethnicity variables: non-Hispanic whites, non-Hispanic blacks, Hispanics, and non-Hispanic other racial/ethnic minorities. Health insurance types of payment, type of admission, rural-urban classification, and comorbidity were included (7, 12, 13). The Elixhauser index was calculated for the comorbidity measure, using the International Classification of Disease, Tenth Revision, Clinical Modification (ICD-10 CM) diagnosis code.

### Statistical Analysis

In the descriptive analysis, the patients' characteristics and the bivariate relationship between the patients' clinical outcomes and key demographic covariates, such as age, sex, and race/ethnicity, were examined. Graphical descriptions of the quarterly trends in LOS, ICU admission, and in-hospital death by the key covariates were created. After an unadjusted Poisson regression model was fitted for key outcomes, multivariable models were run to estimate the adjusted risk ratios (aRR), accounting for secondary covariates, including the type of admission, health insurance type as a payment method, urban-rural classification, and provider and quarter fixed effect. Subsequent regression models further controlled for patients' comorbidity. Analysis was also conducted for the association between key outcomes and age, sex, and race/ethnicity, stratified by each quarter to examine the trend of their relationship over the study period. Additionally, as a sensitivity analysis, multivariable regression was performed for LOS and ICU admission after excluding patients who expired at the hospital. While Poisson regression is a suggested analytic approach for the risk of dichotomous outcomes, the errors of the estimation tend to be overestimated. The variance was rectified using robust standard errors so that adjusted test statistics can be used for the statistical significance of estimates (14, 15). This study used public-use hospital discharge data released from the Texas Department of State Health Services, and the information in the database could not be identified. Therefore, institutional review board approval was not required for the present study based on the US Title 45 Code of Federal Regulations, Part 46. All tests were two-tailed, and the statistical significance was set to *P* < 0.05. All analyses were performed using the R statistical software (version 4.1.2).

## Results

### Patient Characteristics

Of a total 98,879 patients with COVID-19, males accounted for 52.3% as shown in Table 1. The age distribution was as follows: 31.9% for the 65–79 age group, 29.6% for those aged 50–64, 16.3% for those aged 80 or older, 16.0% for ages 35–49, 5.1% for ages 20–34, and 1.1% for those aged 19 and below. Whites constituted the largest proportion (42.6%), followed by Hispanics (36.2%), Blacks (13.1%), and other minorities (8.2%). More than half of the patients were covered by private insurance (51.2%); and the rest were covered by Medicare (32.7%), Medicaid (4.3%), and other sources (11.8%). Most patients were admitted through emergency (80.4%) and were from various metro areas, such as large central (39.0%), large fringe (14.9%), and medium (19.9%).

**Table 1:** Characteristics of patients hospitalized with COVID-19.

**Variable**	***N***	**% or mean (sd)**
	98,879	100
**Age**		
≤ 19	1,114	1.1
20–34	5,031	5.1
35–49	15,821	16.0
50–64	29,273	29.6
65–79	31,507	31.9
≥80	16,133	16.3
**Sex**		
Male	47,127	52.3
Female	51,752	47.7
**Race**		
White	42,088	42.6
Black	12,960	13.1
Hispanic	35,751	36.2
Other	8,080	8.2
**Insurance**		
Private	50,628	51.2
Medicare	32,343	32.7
Medicaid	4,210	4.3
Other	11,698	11.8
**Type of admission**		
Emergency	79,537	80.4
Urgent	11,980	12.1
Elective	7,079	7.2
Other	283	0.3
**Urban-Rural classification**		
Large central metro	38,559	39.0
Large fringe metro	14,764	14.9
Medium metro	19,641	19.9
Small metro	8,681	8.8
Micropolitan	9,635	9.7
Non-core	7,599	7.7
**Quarter**		
2nd quarter	13,202	13.4
3rd quarter	37,492	37.9
4th quarter	48,185	48.7
Comorbidity	98,879	3.4 (2.0)

### Descriptive LOS, ICU Admission, and In-hospital Death

**Figure 1:**
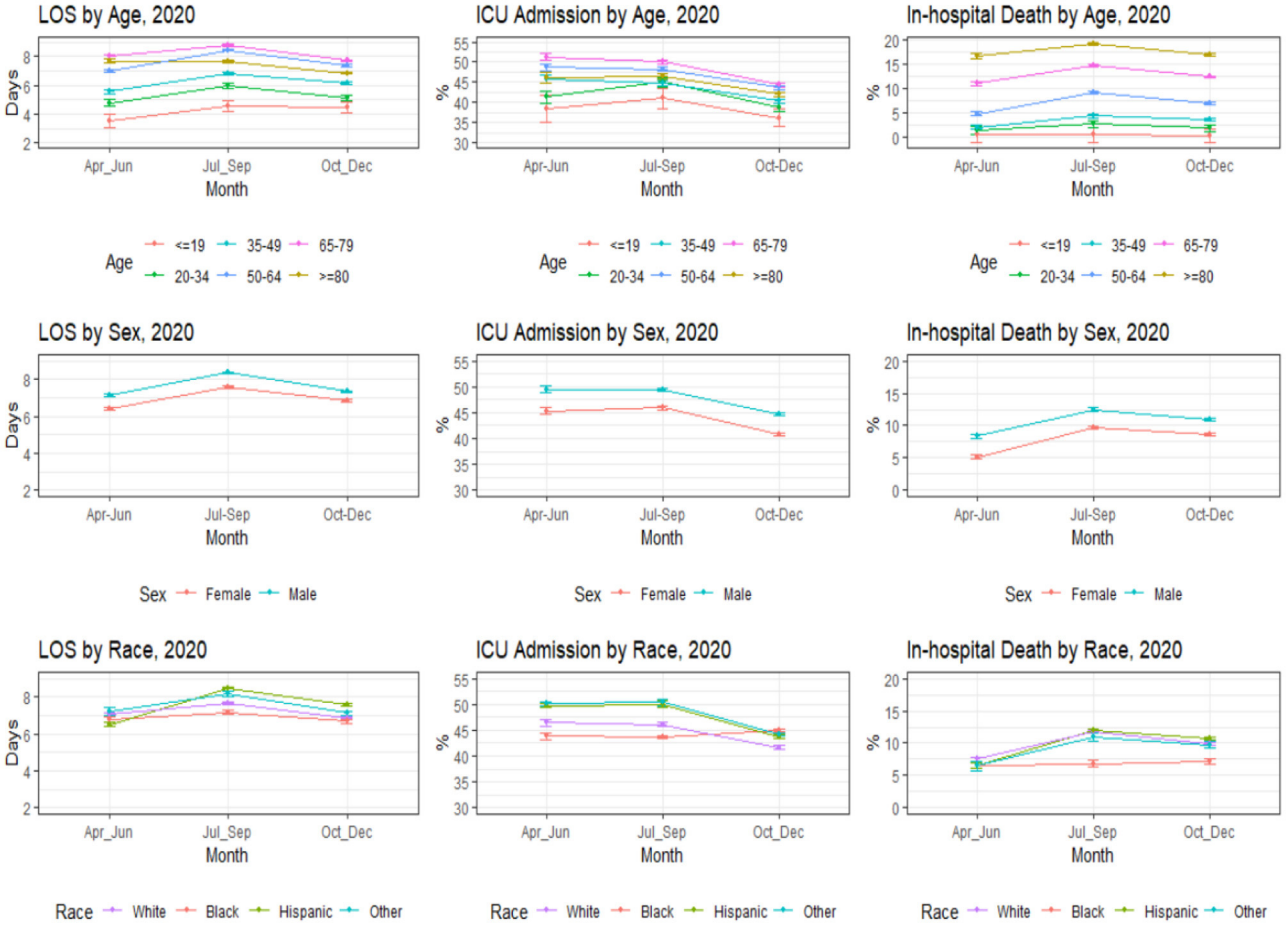
Trends in LOS, ICU admission, and In-hospital death by age, sex, and race/ethnicity.

The bivariate analysis revealed that the patients' mean LOS was 7.4 days (sd 7.9), and the median LOS was 5 days (Table 2). About 45.3% of the patients were admitted to the ICU, and 9.9% expired at the hospital. Hispanics (10.7%), whites (10.3%), and other racial minorities (9.7%) had higher in-hospital death rates than blacks (6.9%). Older adults, particularly those aged 80 and over (17.6%) and those aged 65–79 (13.0%) had a significantly higher in-hospital death rate than patients below 20 years old (0.4%). LOS, ICU admission, and in-hospital death rates across demographic characteristics were largely consistent over the last three quarters of 2020 (Figure 1).

**Table 2:** Summary of LOS, ICU admission, and in-hospital death by age, sex, and race/ethnicity among patients hospitalized with COVID-19.

	**LOS (days)**	**ICU admission**	**In-hospital death**
	**Mean (sd)/ Median**	**%**	**%**
Overall	7.4 (7.9) / 5	45.3	9.9
**Age**			
≤ 19	4.3 (6.0) / 3	38.5	0.4
20–34	5.4 (6.4) / 4	41.8	2.0
35–49	6.3 (7.3) / 4	43.1	3.6
50–64	7.7 (8.8) / 5	46.2	7.5
65–79	8.1 (8.2) / 5	47.2	13.0
≥80	7.2 (6.4) / 5	44.0	17.6
**Sex**			
Male	7.7 (8.2)/ 5	47.2	11.1
Female	7.1 (7.6)/ 5	43.4	8.6
**Race**			
White	7.2 (7.4)/ 5	43.6	10.3
Black	6.9 (7.5)/ 5	44.3	6.9
Hispanic	7.8 (8.5)/ 5	47.4	10.7
Other	7.5 (8.2)/ 5	47.5	9.7

### Differences in LOS From Multivariable Analysis

**Figure 2:**
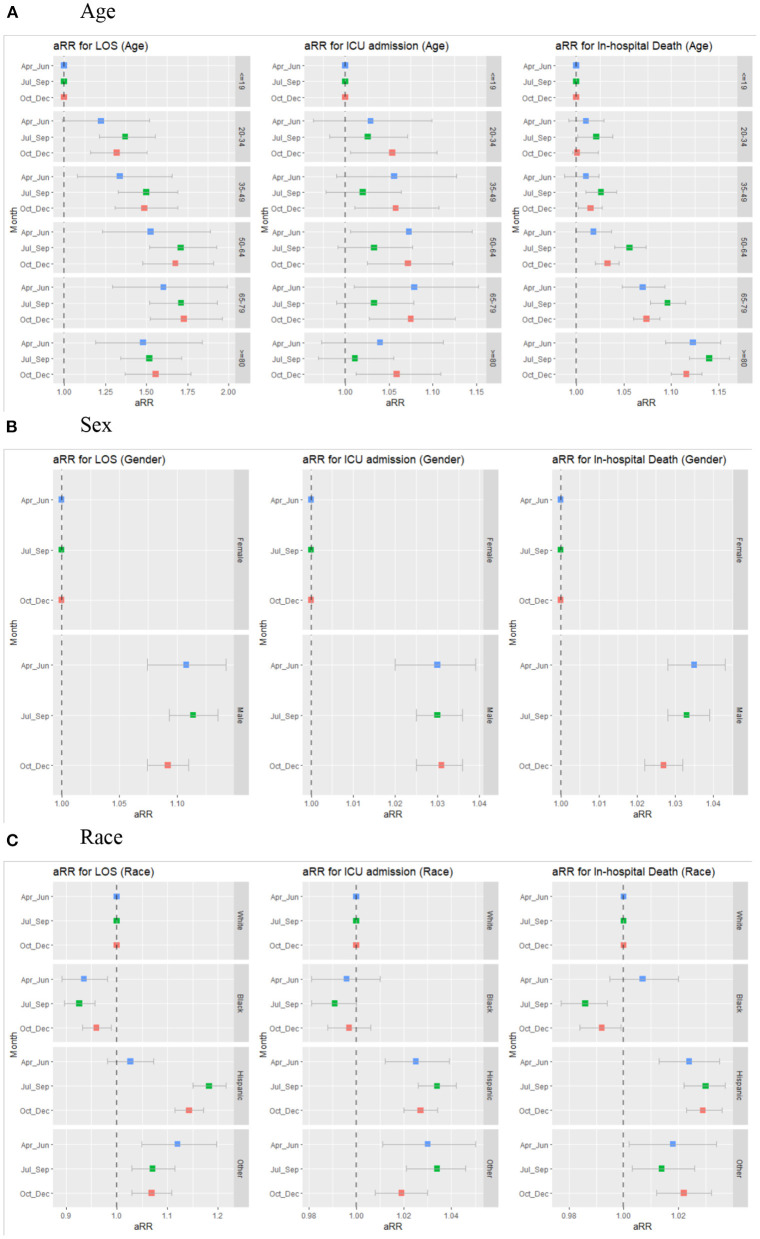
Trends in adjusted risk ratios for LOS, ICU admission, and In-hospital death by age, sex, race/ethnicity over the last three quarters of 2020. **(A)** Age. **(B)** Sex. **(C)** Race. The estimates are from multivariable regression analysis stratified by each quarter, adjusting for covariates in Model 1 and comorbidity of patients.

Compared to the youngest group (those aged 19 and below), patients, including those aged 65–79 (aRR 1.73, 95%CI 1.60–1.88; *p* < 0.000), 50–64 (aRR 1.70, 95%CI 1.57–1.84; *p* < 0.000), 80 and over (1.56, 95%CI 1.43–1.69; *p* < 0.000) all had longer LOS in **Table 4**. Males showed extended LOS relative to females (aRR 1.10, 95%CI 1.09–1.12; *p* < 0.000). Hispanics (aRR 1.14, 95%CI 1.12–1.16; *p* < 0.000) and other racial minorities (aRR 1.09, 95%CI 1.06–1.12; *p* < 0.000) had longer hospital stays, but blacks had shorter hospital stays (aRR 0.94, 95%CI 0.93–0.96; *p* < 0.000) than whites. From April through June, the LOS of Hispanics did not differ significantly from that of whites. However, in later months, Hispanics had a significantly longer LOS than whites, whereas blacks consistently showed a shorter LOS than whites (Figure 2 and Supplementary Table 1). The variation in LOS between males and females slightly decreased in the fourth quarter, while the variations in age remained mostly the same.

### Differences in ICU Admission From Multivariable Analysis

The multivariable analysis showed that those aged 35–49 (aRR 1.04, 95%CI 1.01–1.07; *p* ≤ 0.012), 50–64 (aRR 1.05, 95%CI, 1.02–1.08; *p* = 0.001), 65–79 (aRR 1.05, 95%CI 1.02–1.08; *p* < 0.000), and 80 and over (aRR 1.03, 95%CI 1.01–1.06; *p* = 0.040) had an increased risk of ICU admission compared to younger patients (**Table 4** and Supplementary Table 2). While other age groups compared to patients <19 years old did not show differences in the first two quarters, they had significantly higher ICU admission in the fourth quarter. Over the three quarters, Hispanics and other minorities had consistently higher ICU admission rates than their white counterparts (Figure 2). Males experienced more frequent ICU admissions than females (aRR 1.03, 95%CI 1.03–1.04; *p* < 0.000), and this pattern remained over time.

### Differences in In-hospital Death From Multivariable Analysis

Consistent with the unadjusted model (Table 3), the adjusted analysis demonstrated that the oldest group (those aged 80 and over) had the highest risk of in-hospital death (aRR 1.13, 95%CI 1.11–1.14; *p* < 0.000). The 65–79 (aRR 1.08, 95%CI 1.07–1.09; *p* < 0.000) and 50–64 (aRR 1.04, 95%CI 1.03–1.05; *p* < 0.000) age groups also suffered higher mortality rates than those aged 19 and below (Table 4 and Supplementary Table 3). These age differences in in-hospital deaths continued over time, with only slight changes (Figure 2). Males had a higher in-hospital death rate than females (aRR 1.03, 95%CI 1.02–1.03) although the difference slightly diminished over the three quarters. Hispanics (aRR 1.03, 95%CI 1.02–1.03; *p* < 0.000) and other minorities (aRR 1.02, 95%CI 1.01–1.03; *p* < 0.000) exhibited higher in-hospital death rates than whites, and the differences remained over quarters. In contrast, blacks had a lower in-hospital death rate than whites in later quarters. The results from sensitivity analysis with exclusion of patients who expired at the hospital were similar to the primary results (Supplementary Table 4).

**Table 3:** Unadjusted association between key outcomes and age, sex, and race.

	**Unadjusted**					
	**LOS**		**ICU admission**		**Death**	
	**RR (95%CI)**	***p***	**RR (95%CI)**	***p***	**RR (95%CI)**	***p***
**Age**						
≤ 19	Ref.		Ref.		Ref.	
20–34	1.25 (1.14,1.36)	<0.000	1.02 (1.00,1.05)	0.044	1.02 (1.01,1.02)	<0.000
35–49	1.46 (1.34,1.58)	<0.000	1.03 (1.01,1.06)	0.003	1.03 (1.03,1.04)	<0.000
50–64	1.79 (1.65,1.94)	<0.000	1.06 (1.03,1.08)	<0.000	1.07 (1.07,1.08)	<0.000
65–79	1.88 (1.73,2.04)	<0.000	1.06 (1.04,1.09)	<0.000	1.13 (1.12,1.13)	<0.000
≥80	1.67 (1.53,1.81)	<0.000	1.04 (1.02,1.06)	<0.000	1.17 (1.16,1.18)	<0.000
**Sex**						
Female	Ref.		Ref.		Ref.	
Male	1.09 (1.07,1.10)		1.03 (1.02,1.03)	<0.000	1.02 (1.02,1.03)	<0.000
**Race**						
White	Ref.		Ref.		Ref.	
Black	0.96 (0.94,0.98)	0.001	1.00 (1.00,1.01)	0.162	0.97 (0.96,0.97)	<0.000
Hispanic	1.09 (1.08,1.11)	<0.000	1.03 (1.02,1.03)	<0.000	1.00 (1.00,1.01)	0.082
Other	1.06 (1.03,1.08)	<0.000	1.03 (1.02,1.04)	<0.000	0.99 (0.99,1.00)	0.124

**Table 4:** Adjusted association between key outcomes and age, sex, and race.

	**Model 1**						**Model 2 (Model 1 + comorbidity)**					
	**LOS**		**ICU admission**		**Death**		**LOS**		**ICU admission**		**Death**	
	**RR (95%CI)**	***p***	**RR (95%CI)**	***p***	**RR (95%CI)**	***p***	**aRR (95%CI)**	***p***	**aRR (95%CI)**	***p***	**aRR (95%CI)**	***P***
**Age**												
≤ 19	Ref.		Ref.		Ref.		Ref.		Ref.		Ref.	
20–34	1.54 (1.40, 1.70)	<0.000	1.06 (1.03, 1.09)	<0.000	1.05 (1.04, 1.06)	<0.000	1.34 (1.24, 1.46)	<0.000	1.03 (1.00, 1.06)	0.033	1.01 (1.01, 1.02)	0.002
35–49	1.81 (1.65, 1.99)	<0.000	1.08 (1.05, 1.11)	<0.000	1.06 (1.05, 1.07)	<0.000	1.49 (1.38, 1.62)	<0.000	1.04 (1.01, 1.07)	0.012	1.02 (1.01, 1.03)	<0.000
50–64	2.23 (2.03, 2.44)	<0.000	1.11 (1.08, 1.15)	<0.000	1.10 (1.09, 1.11)	<0.000	1.70 (1.57, 1.84)	<0.000	1.05 (1.02, 1.08)	0.001	1.04 (1.03, 1.05)	<0.000
65–79	2.41 (2.19, 2.64)	<0.000	1.13 (1.10, 1.16)	<0.000	1.16 (1.15, 1.17)	<0.000	1.73 (1.60, 1.88)	<0.000	1.05 (1.02, 1.08)	<0.000	1.08 (1.07, 1.09)	<0.000
≥80	2.22 (2.02, 2.43)	<0.000	1.11 (1.08, 1.15)	<0.000	1.22 (1.21, 1.23)	<0.000	1.56 (1.43, 1.69)	<0.000	1.03 (1.01, 1.06)	0.040	1.13 (1.11, 1.14)	<0.000
**Sex**												
Female	Ref.		Ref.		Ref.		Ref.		Ref.		Ref.	
Male	1.08 (1.06, 1.09)	<0.000	1.02 (1.02, 1.03)	<0.000	1.03 (1.02, 1.03)	<0.000	1.10 (1.09, 1.12)	<0.000	1.03 (1.03, 1.04)	<0.000	1.03 (1.02, 1.03)	<0.000
**Race**												
White	Ref.		Ref.		Ref.		Ref.		Ref.		Ref.	
Black	0.99 (0.97, 1.02)	0.621	1.00 (1.00, 1.01)	0.621	1.00 (1.00, 1.01)	0.394	0.94 (0.93, 0.96)	<0.000	0.99 (0.99, 1.00)	0.051	0.99 (0.98, 1.00)	0.004
Hispanic	1.12 (1.10, 1.14)	<0.000	1.02 (1.02, 1.02)	<0.000	1.02 (1.02, 1.03)	<0.000	1.14 (1.12,1.16)	<0.000	1.03 (1.02, 1.03)	<0.000	1.03 (1.02, 1.03)	<0.000
Other	1.05 (1.03, 1.08)	<0.000	1.02 (1.01, 1.03)	<0.000	1.01 (1.00, 1.02)	0.002	1.09 (1.06,1.12)	<0.000	1.03 (1.02, 1.03)	<0.000	1.02 (1.01, 1.03)	<0.000

## Discussion

This study examined age, sex, and racial/ethnic differences in LOS, ICU admission, and in-hospital death among patients hospitalized with COVID-19. Overall, ICU admission decreased over time, consistent with previous reports (16). In contrast, rates of LOS and in-hospital mortality remained over the study period. The study findings showed that the assessed demographic characteristics were important predictors of LOS, ICU admission, and in-hospital death, and these associations were largely consistent throughout 2020.

Earlier investigations reported significant variations in health outcomes between age groups during the COVID-19 pandemic (2, 7). Consistent with previous findings, this study found that age was a strong predictor of higher mortality and ICU admission rates as well as longer hospital stays. While the overall ICU admission rate had decreased over time as revealed in the descriptive analysis, the adjusted analysis showed significant differences between age groups. Patients aged 80 and over and those aged 65–79 had the highest mortality rates; this pattern persisted from April to December 2020. The adjusted ICU admission rate was also significantly higher among the older age groups (ages 50–64, 65–79, and 80 and over), similar to the findings in previous studies, suggesting severe conditions disproportionally among older adults (3, 17).

The COVID-19 pandemic has shed light on racial health disparities. This study found that Hispanics and other racial minorities including Asian and Pacific Islanders had an increased risk of ICU admission and in-hospital mortality compared to whites, similar to earlier findings (8, 11, 18, 19). When stratified by quarters, the present study found continued racial variations in assessed health outcomes. These persistent disparities were also reported in multiple studies (8, 20); They suggested higher ICU admission and in-hospital mortality in Hispanics and Asian or Pacific Islanders over time. Although another study tended to show an increased risk in the assessed outcomes among racial minorities compared to whites over a period of time, the results were not statistically significant (16). While some variations across studies exist, the findings of the present study using a large database of the inpatient population strengthen the knowledge base and highlight significant health disparities among Hispanics and minorities (8, 11, 18). Moreover, there was also evidence of a significantly higher risk of prolonged hospital stays among Hispanic subgroups from the third quarter of 2020 in this study. The soaring risk of lengthy hospital stays in this group relative to the white group in later quarters may be driven by a lack of early testing and diagnosis of the coronavirus, leading to rapid deterioration of health conditions and so high mortality later (21). Markedly, Hispanic patients were the most vulnerable to in-hospital mortality and the intensified risk of death continued throughout the year. Although reasons for the poor clinical health outcomes are to be further explored in terms of both socio-economic or environmental and physiological factors, preexisting social and health inequities that a historically underserved minorities experience may have contributed to severe health conditions associated with COVID-19 (19, 22).

Conversely, blacks tended to show a lower likelihood of ICU admission and in-hospital mortality compared to their white counterparts. Overall, the findings of the present study are comparable with what has been reported from previous studies conducted in health care settings—blacks had either a lower or similar risk of ICU admission and hospital mortality compared to whites (7, 12, 16, 22). Although one study found higher ICU admission among blacks compared to whites, this pattern did not remain when the analysis was stratified by a certain time period (16). Notably, these findings contradict those from studies of the general population that showed more severe conditions in black persons (7, 8). The contrasting results between hospitalized patients and general populations may attribute to a number of factors. A lower or similar risk of severe conditions among black persons in the general population may be due to barriers to access to health care that blacks experience because of either a lack of insurance or underinsurance (23). Their poor access may cause exacerbated health conditions and increased deaths outside the hospital and, therefore, a higher overall mortality rate. On the other hand, undiagnosed cases due to asymptomatic infections and delayed diagnosis may lead to severe health conditions and later be recognized as deaths caused by COVID-19 (24). The findings of this study with those of previous studies suggest that, while the poor health outcomes are more marked among blacks outside of a hospital, once admitted, blacks may have an equal or lower likelihood of experiencing severe conditions compared to whites (12).

This study found that males had an increased risk of longer hospital stays, ICU admission, and in-hospital death compared to females. These results are consistent with prior reports on both hospitalized patients and the general population (7, 8, 25). Despite the slight decrease over time, these sex differences in the assessed clinical outcomes largely remained. While the drivers of these differences are still uncertain, the varying clinical outcomes might be driven by behavioral differences between males and females, such as the higher prevalence of smoking and drinking among males (5). Furthermore, biological pathways and immune responses have been suggested as likely explanations for the significant sex differences in clinical outcomes associated with COVID-19 (26, 27).

The study has several limitations. First, given the nature of the observational study, unmeasured patient information may remain. Also, as the database used for this study included mainly patients' data associated with hospitalization, unobserved information with respect to the cross-hospital variations may exist and confound the observed association between exposure and outcome. Although the multivariable analysis that adjusted for study covariates and a provider identifier as fixed effects would improve the unmeasured issues, the potential bias due to unmeasured confounding may still affect the estimation and undermine the study findings. Second, one of the strengths of the present study is the inclusion of all state-licensed hospitals in Texas with a few exceptions, which provides more robust and representative evidence of health outcomes among the inpatient population hospitalized with COVID-19. However, given the Texas context, a caution is still needed in interpreting the study findings in other contexts. Third, despite the adjusted regression models being performed to control for various patient characteristics, the analysis was not designed to assess causality given the nature of the observational study. Fourth, while analysis using months rather than quarters is more desirable, the study did not examine monthly analysis as only quarter indicators were available. Despite several limitations, this study documents important evidence of differential risks in clinical outcomes associated with COVID-19 across patient demographics.

## Conclusions

The COVID-19 pandemic is one of the most disturbing public health challenges in the history of human disease. This study revealed the trend of clinical outcomes associated with COVID-19, showing population subgroups, such as older adults, males, and racial/ethnic minorities, disproportionately affected. The pandemic has raised our awareness not only of the danger of infectious disease but also of the amplified health disparities. While the nation has to continue practicing public health measures to minimize the harm caused by the novel virus and its variants, serious consideration must be given to improving the healthcare and health of the marginalized and vulnerable populations during and beyond the pandemic.

## Data Availability Statement

Publicly available datasets were analyzed in this study. This data can be found here: The de-identified datasets for this study can be obtained from the Texas Health and Human Services https://www.dshs.texas.gov/thcic/hospitals/Inpatientpudf.shtm.

## Author Contributions

The author confirms being the sole contributor of this work and has approved it for publication.

## Conflict of Interest

The author declares that the research was conducted in the absence of any commercial or financial relationships that could be construed as a potential conflict of interest.

## Publisher's Note

All claims expressed in this article are solely those of the authors and do not necessarily represent those of their affiliated organizations, or those of the publisher, the editors and the reviewers. Any product that may be evaluated in this article, or claim that may be made by its manufacturer, is not guaranteed or endorsed by the publisher.
