# Supplementary material for: Age, Sex, and Race/Ethnicity in Clinical Outcomes Among Patients Hospitalized With COVID-19, 2020
Source: Front Med (Lausanne). 2022 May 12;9:850536. doi: 10.3389/fmed.2022.850536 (PMC9133563; doi:10.3389/fmed.2022.850536)
Supplement: Supplementary file 1 [file Data_Sheet_1.docx]

Supplements

Table 1. Adjusted association between LOS and age, sex, and race/ethnicity

|  | April-June | | | | July-September | | | | October-December | | | | |
| --- | --- | --- | --- | --- | --- | --- | --- | --- | --- | --- | --- | --- | --- |
|  | Model 1 | | Model 2  (Model 1 + comorbidity) | | Model 1 | | Model 2  (Model 1 + comorbidity) | | Model 1 | | Model 2  (Model 1 + comorbidity) | | |
|  | aRR (95%CI) | p | aRR (95%CI) | P | aRR (95%CI) | p | aRR (95%CI) | p | aRR (95%CI) | p | aRR (95%CI) | p |  |
| **Age** |  |  |  |  |  |  |  |  |  |  |  |  |  |
| <=19 | Ref. |  | Ref. |  | Ref. |  | Ref. |  | Ref. |  | Ref. |  |  |
| 20-34 | 1.42 (1.13, 1.77) | 0.002 | 1.22 (0.99, 1.52) | 0.066 | 1.62 (1.41, 1.86) | <.000 | 1.37 (1.21, 1.55) | <.000 | 1.49 (1.27, 1.75) | <.000 | 1.32 (1.16, 1.50) | <.000 |  |
| 35-49 | 1.65 (1.33, 2.06) | <.000 | 1.34 (1.08, 1.66) | 0.007 | 1.86 (1.62, 2.13) | <.000 | 1.50 (1.33, 1.69) | <.000 | 1.79 (1.53, 2.09) | <.000 | 1.49 (1.31, 1.69) | <.000 |  |
| 50-64 | 2.04 (1.64, 2.54) | <.000 | 1.53 (1.23, 1.89) | <.000 | 2.30 (2.01, 2.63) | <.000 | 1.71 (1.52, 1.93) | <.000 | 2.15 (1.84, 2.50) | <.000 | 1.68 (1.48, 1.91) | <.000 |  |
| 65-79 | 2.31 (1.85, 2.88) | <.000 | 1.60 (1.29, 1.99) | <.000 | 2.44 (2.14, 2.79) | <.000 | 1.71 (1.52, 1.93) | <.000 | 2.33 (2.00, 2.72) | <.000 | 1.73 (1.52, 1.96) | <.000 |  |
| >=80 | 2.19 (1.75, 2.73) | <.000 | 1.48 (1.19, 1.84) | <.000 | 2.21 (1.93, 2.53) | <.000 | 1.52 (1.34, 1.71) | <.000 | 2.15 (1.84, 2.51) | <.000 | 1.56 (1.37, 1.77) | <.000 |  |
| **Sex** |  |  |  |  |  |  |  |  |  |  |  |  |  |
| Female | Ref. |  | Ref. |  | Ref. |  | Ref. |  | Ref. |  | Ref. |  |  |
| Male | 1.09 (1.05, 1.12) | <.000 | 1.11 (1.07, 1.14) | <.000 | 1.08 (1.06, 1.11) | <.000 | 1.11 (1.09, 1.14) | <.000 | 1.07 (1.05, 1.09) | <.000 | 1.09 (1.07, 1.11) | <.000 |  |
| **Race** |  |  |  |  |  |  |  |  |  |  |  |  |  |
| White | Ref. |  | Ref. |  | Ref. |  | Ref. |  | Ref. |  | Ref. |  |  |
| Black | 0.97 (0.93, 1.02) | 0.305 | 0.94 (0.89, 0.98) | 0.007 | 0.97 (0.94, 1.08) | 0.128 | 0.93 (0.90, 0.96) | <.000 | 1.01 (0.98. 1.05) | 0.377 | 0.96 (0.93, 0.99) | 0.008 |  |
| Hispanic | 0.99 (0.94, 1.03) | 0.541 | 1.03 (0.98, 1.07) | 0.245 | 1.15 (1.12, 1.18) | 0.112 | 1.18 (1.15, 1.21) | <.000 | 1.13 (1.10, 1.16) | <.000 | 1.14 (1.11, 1.17) | <.000 |  |
| Other | 1.08 (1.01, 1.16) | <.000 | 1.12 (1.05, 1.20) | 0.001 | 1.03 (0.99, 1.08) | <.000 | 1.07 (1.03, 1.11) | 0.001 | 1.03 (0.99, 1.07) | 0.086 | 1.07 (1.03, 1.11) | <.000 |  |

Note: Model 1 accounted for type of admission, urban-rural classification, and hospital and quarter fixed effects. Model 2 further controlled for comorbidity of patients in addition to covariates in Model 1.

Table 2. Adjusted association between ICU admission and age, sex, and race/ethnicity

|  | April-June | | | | July-September | | | | October-December | | | | |
| --- | --- | --- | --- | --- | --- | --- | --- | --- | --- | --- | --- | --- | --- |
|  | Model 1 | | Model 2  (Model 1 + comorbidity) | | Model 1 | | Model 2  (Model 1 + comorbidity) | | Model 1 | | Model 2  (Model 1 + comorbidity) | | |
|  | aRR (95%CI) | p | aRR (95%CI) | p | aRR (95%CI) | p | aRR (95%CI) | p | aRR (95%CI) | p | aRR (95%CI) | p |  |
| **Age** |  |  |  |  |  |  |  |  |  |  |  |  |  |
| <=19 | Ref. |  | Ref. |  | Ref. |  | Ref. |  | Ref. |  | Ref. |  |  |
| 20-34 | 1.06 (0.99, 1.13) | 0.080 | 1.03 (0.96, 1.10) | 0.402 | 1.06 (1.01, 1.11) | 0.012 | 1.03 (0.98, 1.07) | 0.250 | 1.09 (1.04, 1.14) | 0.001 | 1.05 (1.00, 1.11) | 0.027 |  |
| 35-49 | 1.10 (1.03, 1.18) | 0.003 | 1.06 (0.99, 1.13) | 0.099 | 1.06 (1.02, 1.11) | 0.004 | 1.02 (0.98, 1.06) | 0.355 | 1.11 (1.06, 1.16) | <.000 | 1.06 (1.01, 1.11) | 0.016 |  |
| 50-64 | 1.14 (1.07, 1.22) | <.000 | 1.07 (1.01, 1.15) | 0.033 | 1.10 (1.05, 1.14) | <.000 | 1.03 (0.99, 1.08) | 0.126 | 1.14 (1.09, 1.19) | <.000 | 1.07 (1.03, 1.12) | 0.003 |  |
| 65-79 | 1.17 (1.09, 1.25) | <.000 | 1.08 (1.01, 1.15) | 0.024 | 1.11 (1.06, 1.16) | <.000 | 1.03 (0.99, 1.08) | 0.130 | 1.16 (1.10, 1.21) | <.000 | 1.08 (1.03, 1.13) | 0.002 |  |
| >=80 | 1.13 (1.06, 1.21) | <.000 | 1.04 (0.97, 1.11) | 0.243 | 1.09 (1.05, 1.14) | <.000 | 1.01 (0.97, 1.06) | 0.617 | 1.15 (1.09, 1.20) | <.000 | 1.06 (1.01, 1.11) | 0.014 |  |
| **Sex** |  |  |  |  |  |  |  |  |  |  |  |  |  |
| Female | Ref. |  | Ref. |  | Ref. |  | Ref. |  | Ref. |  | Ref. |  |  |
| Male | 1.02 (1.01, 1.03) | <.000 | 1.03 (1.02, 1.04) | <.000 | 1.02 (1.02, 1.03) | <.000 | 1.03 (1.02, 1.04) | <.000 | 1.02 (1.02, 1.03) | <.000 | 1.03 (1.03, 1.04) | <.000 |  |
| **Race** |  |  |  |  |  |  |  |  |  |  |  |  |  |
| White | Ref. |  | Ref. |  | Ref. |  | Ref. |  | Ref. |  | Ref. |  |  |
| Black | 1.00 (0.99, 1.02) | 0.793 | 1.00 (0.98, 1.01) | 0.549 | 1.00 (0.99, 1.01) | 0.962 | 0.99 (0.98, 1.00) | 0.062 | 1.01 (1.00, 1.02) | 0.068 | 1.00 (0.99, 1.01) | 0.515 |  |
| Hispanic | 1.02 (1.00, 1.03) | 0.013 | 1.03 (1.01, 1.04) | <.000 | 1.03 (1.02, 1.04) | <.000 | 1.03 (1.03, 1.04) | <.000 | 1.02 (1.02, 1.03) | <.000 | 1.03 (1.02, 1.03) | <.000 |  |
| Other | 1.02 (1.00, 1.04) | 0.021 | 1.03 (1.01, 1.05) | 0.002 | 1.03 (1.01, 1.04) | <.000 | 1.03 (1.02, 1.05) | <.000 | 1.01 (1.00, 1.02) | 0.070 | 1.02 (1.01, 1.03) | <.000 |  |

Note: Model 1 accounted for type of admission, urban-rural classification, and hospital and quarter fixed effects. Model 2 further controlled for comorbidity of patients in addition to covariates in Model 1.

Table 3. Adjusted association between In-hospital Death and age, sex, and race/ethnicity

|  | April-June | | | | July-September | | | | October-December | | | |
| --- | --- | --- | --- | --- | --- | --- | --- | --- | --- | --- | --- | --- |
|  | Model 1 | | Model 2 | | Model 1 | | Model 2 | | Model 1 | | Model 2 | |
|  | aRR (95%CI) | p | aRR (95%CI) | p | aRR (95%CI) | p | aRR (95%CI) | p | aRR (95%CI) | p | aRR (95%CI) | p |
| **Age** |  |  |  |  |  |  |  |  |  |  |  |  |
| <=19 | Ref. |  | Ref. |  | Ref |  | Ref. |  | Ref. |  | Ref. |  |
| 20-34 | 1.04 (1.02, 1.06) | <.000 | 1.01 (0.99, 1.03) | 0.286 | 1.06 (1.04, 1.07) | <.000 | 1.02 (1.00, 1.04) | 0.010 | 1.04 (1.03, 1.05) | <.000 | 1.00 (1.00, 1.02) | 0.158 |
| 35-49 | 1.05 (1.03, 1.06) | <.000 | 1.01 (0.99, 1.02) | 0.553 | 1.07 (1.06, 1.09) | <.000 | 1.03 (1.01, 1.04) | 0.002 | 1.06 (1.05, 1.07) | <.000 | 1.02 (1.00, 1.03) | 0.020 |
| 50-64 | 1.07 (1.05, 1.09) | <.000 | 1.02 (1.00, 1.04) | 0.066 | 1.12 (1.11, 1.14) | <.000 | 1.06 (1.04, 1.07) | <.000 | 1.09 (1.08, 1.11) | <.000 | 1.03 (1.02, 1.05) | <.000 |
| 65-79 | 1.14 (1.12, 1.17) | <.000 | 1.07 (1.05, 1.09) | <.000 | 1.18 (1.16, 1.20) | <.000 | 1.10 (1.08, 1.12) | <.000 | 1.15 (1.14, 1.17) | <.000 | 1.07 (1.06, 1.09) | <.000 |
| >=80 | 1.21 (1.18, 1.24) | <.000 | 1.23 (1.09, 1.15) | <.000 | 1.24 (1.21, 1.26) | <.000 | 1.14 (1.12, 1.16) | <.000 | 1.21 (1.19, 1.22) | <.000 | 1.12 (1.10, 1.13) | <.000 |
| **Sex** |  |  |  |  |  |  |  |  |  |  |  |  |
| Female | Ref. |  | Ref. |  | Ref |  | Ref. |  | Ref. |  | Ref. |  |
| Male | 1.03 (1.02, 1.04) | <.000 | 1.04 (1.03, 1.04) | <.000 | 1.03 (1.02, 1.03) | <.000 | 1.03 (1.02, 1.04) | <.000 | 1.02 (1.01, 1.03) | <.000 | 1.03 (1.02, 1.03) | <.000 |
| **Race** |  |  |  |  |  |  |  |  |  |  |  |  |
| White | Ref. |  | Ref. |  | Ref |  | Ref. |  | Ref. |  | Ref. |  |
| Black | 1.01 (1.00, 1.03) | 0.042 | 1.01 (0.99, 1.02) | 0.261 | 1.00 (0.99, 1.00) | 0.253 | 0.99 (0.98, 0.99) | 0.001 | 1.00 (1.00, 1.01) | 0.441 | 0.99 (0.98, 1.00) | 0.035 |
| Hispanic | 1.02 (1.01, 1.03) | 0.004 | 1.02 (1.01, 1.04) | <.000 | 1.02 (1.02, 1.03) | <.000 | 1.03 (1.02, 1.04) | <.000 | 1.03 (1.02, 1.03) | <.000 | 1.03 (1.02, 1.04) | <.000 |
| Other | 1.01 (1.00, 1.03) | 0.155 | 1.02 (1.00, 1.03) | 0.023 | 1.01 (0.99, 1.02) | 0.335 | 1.01 (1.00, 1.03) | 0.014 | 1.01 (1.00, 1.02) | 0.008 | 1.02 (1.01, 1.03) | <.000 |

Note: Model 1 accounted for type of admission, urban-rural classification, and hospital and quarter fixed effects. Model 2 further controlled for comorbidity of patients in addition to covariates in Model 1.

Table 4. Adjusted association between key outcomes and age, sex, and race/ethnicity, exclusion of expired patients at discharge

|  | Model 1 | | | | Model 2  (Model 1 + comorbidity) | | | |
| --- | --- | --- | --- | --- | --- | --- | --- | --- |
|  | LOS |  | ICU admission |  | LOS |  | ICU admission |  |
|  | RR (95%CI) | p | RR (95%CI) | p | aRR (95%CI) |  | aRR (95%CI) | p |
| **Age** |  |  |  |  |  |  |  |  |
| <=19 | Ref. |  | Ref. |  | Ref. | p | Ref. |  |
| 20-34 | 1.45 (1.32, 1.59) | <.000 | 1.05 (1.02, 1.08) | 0.001 | 1.28 (1.18, 1.40) | <.000 | 1.03 (1.00, 1.06) | 0.058 |
| 35-49 | 1.66 (1.51, 1.82) | <.000 | 1.07 (1.04, 1.10) | <.000 | 1.40 (1.29, 1.52) | <.000 | 1.03 (1.00, 1.06) | 0.033 |
| 50-64 | 1.95 (1.78, 2.14) | <.000 | 1.09 (1.06, 1.12) | <.000 | 1.55 (1.43, 1.68) | <.000 | 1.04 (1.01, 1.07) | 0.008 |
| 65-79 | 2.07 (1.89, 2.28) | <.000 | 1.09 (1.06, 1.12) | <.000 | 1.56 (1.44, 1.70) | <.000 | 1.03 (1.00, 1.06) | 0.038 |
| >=80 | 2.00 (1.82, 2.20) | <.000 | 1.08 (1.04, 1.11) | <.000 | 1.47 (1.35, 1.59) | <.000 | 1.01 (0.98, 1.04) | 0.418 |
| **Sex** |  |  |  |  |  |  |  |  |
| Female | Ref. |  | Ref. |  | Ref. |  | Ref. |  |
| Male | 1.06 (1.05, 1.07) | <.000 | 1.02 (1.01, 1.02) | <.000 | 1.09 (1.08, 1.10) | <.000 | 1.02 (1.02, 1.03) | <.000 |
| **Race** |  |  |  |  |  |  |  |  |
| White | Ref. |  | Ref. |  | Ref. |  | Ref. |  |
| ack | 0.99 (0.97, 1.01) | 0.316 | 1.00 (0.99, 1.01) | 0.270 | 0.95 (0.93, 0.97) | <.000 | 1.00 (0.99, 1.00) | 0.173 |
| Hispanic | 1.08 (1.06, 1.10) | <.000 | 1.02 (1.01, 1.02) | <.000 | 1.11 (1.09, 1.12) | <.000 | 1.02 (1.01, 1.03) | <.000 |
| Other | 1.05 (1.02, 1.08) | 0.001 | 1.02 (1.01, 1.02) | <.000 | 1.09 (1.06, 1.12) | <.000 | 1.02 (1.02, 1.03) | <.000 |
